# Infectious Diseases Spreading on an Adaptive Metapopulation Network

**DOI:** 10.1109/ACCESS.2020.3016016

**Published:** 2020-08-12

**Authors:** Shanshan Feng, Zhen Jin

**Affiliations:** 1 School of Data Science and TechnologyNorth University of China 66291 Taiyuan 030051 China; 2 Complex Systems Research CenterShanxi University 12441 Taiyuan 030006 China; 3 Shanxi Key Laboratory of Mathematical Techniques and Big Data Analysis on Disease Control and PreventionShanxi University 12441 Taiyuan 030006 China

**Keywords:** Emerging infectious diseases, travel restrictions, adaptive metapopulation network, risk indicator

## Abstract

When an emerging acute infectious disease occurs, travel restrictions, one-way or two-way, are often taken to prevent its global spread. In order to investigate the impact of two-way travel restrictions in the global spread of infectious diseases, this paper defines a risk indicator according to the relative infection density. Based on this risk indicator and an intervention time on two-way travel restrictions, we define an adaptive metapopulation network. Then a susceptible-infectious-removed (SIR) metapopulation model on this network is proposed. The mathematical analysis shows that the basic reproduction number is independent of human mobility. Furthermore, this essay compares the effects of one-way travel restrictions and two-way travel restrictions on the global spread of infectious diseases. It is shown that the adaptive metapopulation network under two-way travel restrictions can effectively suppress the global spread of infectious diseases. We also obtain a threshold of risk indicator to prevent the global spread of infectious diseases by simulations. The earlier the intervention time on two-way travel restriction is, the better to curb the global spread of the disease. Even if two-way travel restrictions are not implemented, controlling the mobility of infectious persons would help prevent the global spread of the disease. This work will throw lights on the prevention and control of the globally spreading of an emerging infectious disease.

## I. Introduction

Whether in the past or at present, infectious diseases have always been serious threats to human life and health. In the 1340s, the black death swept across Europe, killing about 25 million Europeans, a third of the population. From May 2015 to June 2016, the Zika virus spread to 40 countries and territories within the Americas in one year [1]. Furthermore, with the rapid development of globalization, developed transportation makes long-distance travel more and more convenient. However, the mobility of infectious individuals facilitates the global spread of infectious diseases. As of August 9, 2020, the coronavirus disease 2019 (COVID-19) has spread over 215 countries and territories, infected more than 19 million people and caused more than 720,000 deaths. The outbreak of any infectious diseases has a significant impact on humans, either physically, mentally, or economically. Modeling and controlling the global spread of infectious diseases has always been the focus of researches. One of the major models to study the global spread of infectious diseases is a metapopulation network model. For a metapopulation network, each node (subpopulations) represent a country, a city or a town, and individuals in each subpopulation are well-mixed. The mobility of individuals between two connected subpopulations forms the link of the network.

Colizza and Vespignani built heterogeneous mean-field models to describe the transmission of diseases on heterogeneous metapopulation networks under two kinds of mobility patterns: traffic dependent mobility rate and population dependent mobility rate [2]. Their work lays a foundation for future researches on metapopulation networks. From the point of view of network structure, Cao *et al.* studied the rendezvous effects on bipartite metapopulation network and found that rendezvous effects made for the transmission of infectious diseases [3]; Liu *et al.* investigated time-varying metapopulation networks, which slowed down the spread of infectious diseases [4]; Mata *et al.* studied local subpopulation structure on metapopulation networks, that is, individuals within a subpopulation were not well-mixed but within a social network [5]; we defined a second-neighbor network (SNN) and investigated the global spread of infectious disease on a metapopulation network coupled with its SNN [6]. In order to avoid being infected, individuals may respond to infectious diseases. Sandro *et al.* discovered that the adaptive behavior of individuals contributed to the global spreading of diseases [7]. Similar results were obtained in [8]. These two works are based on the same assumption that the higher the relative density of infectious individuals at the destination subpopulation is, the less likely the individuals will travel. In recent years, with the development of big data technology, more and more fine-grained data can be obtained. The metapopulation network model based on these data has achieved great success. Panigutti *et al.* studied recurrent mobility patterns on metapopulaion networks using census data and mobile phone data [9]. Pei *et al.* forecast the spread of influenza in the United States using a metapopulation model [10]. Chinazzi *et al.* used the Global Epidemic and Mobility Model (GLEAM) to forecast the effect of travel limitations on the national and international spread of COVID-19 [11].

When an emerging acute infectious disease occurs, in order to prevent its global spread, each subpopulation (maybe a country, a city, or a town) will assess the risk of disease invasion and timely prevent and control it. One way to prevent the global spread of infectious diseases is to restrict travel, which is a problem of broken links in metapopulation networks. There are two main types of travel restrictions: two-way travel restrictions and one-way travel restrictions. Two-way travel restrictions refer to cutting off the links between the connected subpopulations, with no individuals moving between them. One-way travel restrictions include two types. One is to prevent individuals in a subpopulation from moving into its neighbor subpopulations. The other is to prevent the move-in from individuals in its neighbor subpopulation. Travel restrictions between a subpopulation and its neighbor subpopulations may be on some neighbor subpopulations or on all neighbor subpopulations. City (or country) lockdown is a two-way travel restriction on all neighbor subpopulations. During COVID-19, the municipal government of Wuhan decided to lock down the city on January 23; nation-wide lockdown was implemented on March 10, in Italy; subsequently, Spain, the Czech Republic, France, Belgium announced the nation-wide lockdown.

**FIGURE 1.**
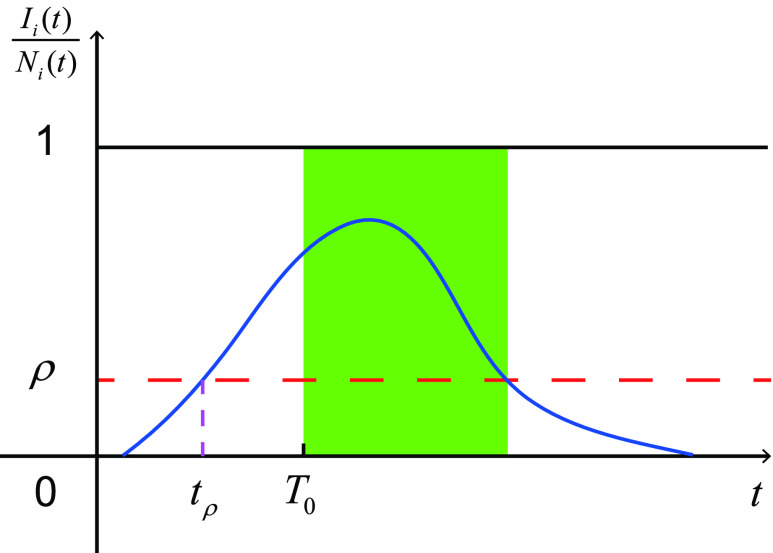
The schematic of the intervention time on two-way travel restriction $T_{0}$. The red dashed line is the risk indicator. The blue line is the time series of the relative infection density of subpopulation $i$, i.e., $I_{i}(t)/N_{i}(t)$. The shaded green area indicates the start and end time of travel restrictions implemented on subpopulation $i$. $t_\rho $ is the minimal time when the subpopulation goes from low risk to high risk.

**FIGURE 2.**
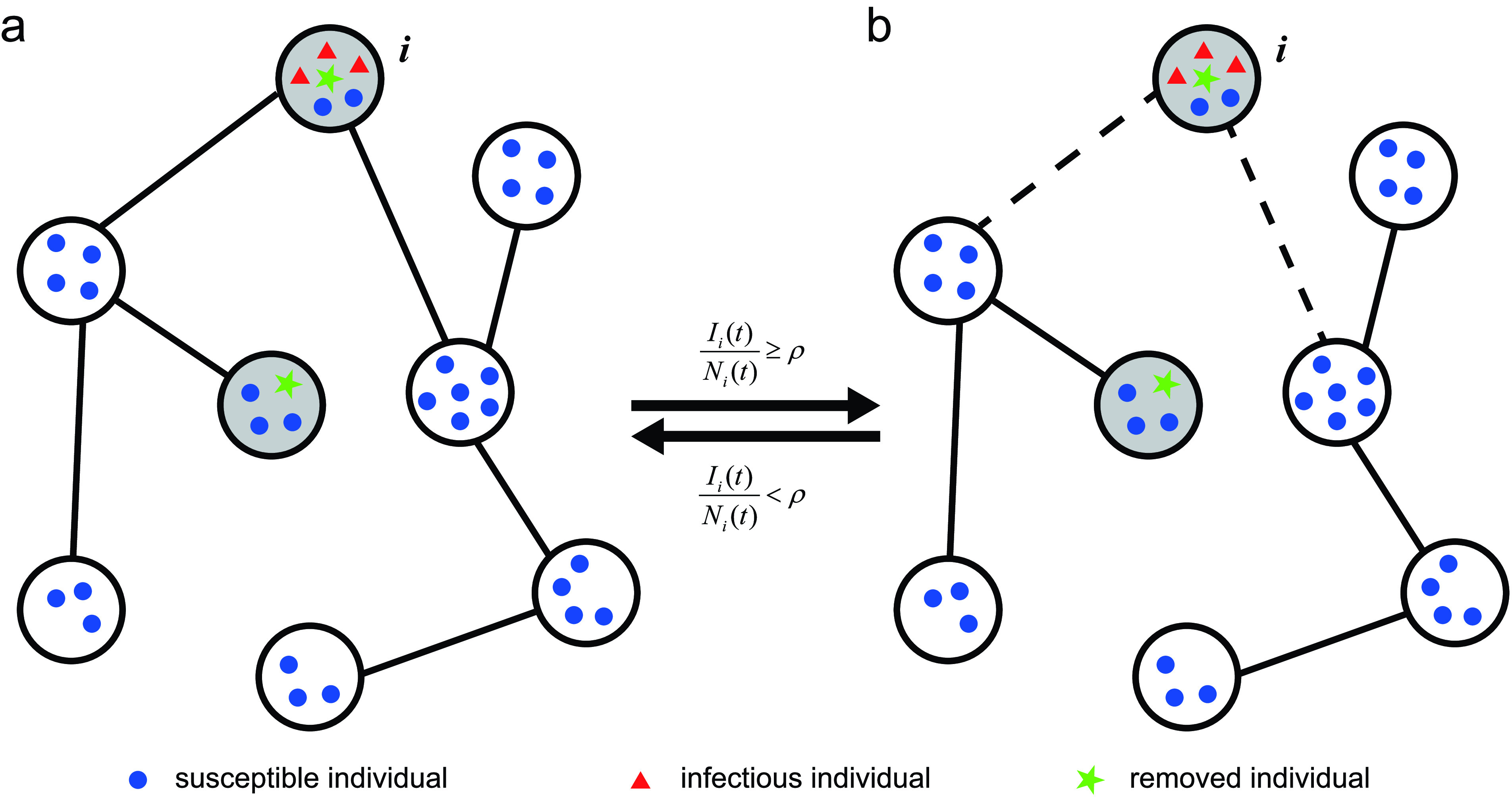
The schematic depiction of an adaptive metapopulation network. Blue circles represent susceptible individuals, red triangles represent infectious individuals, and a green pentagram represents a removed individual. The dashed lines mean that there is no mobility of individuals between the two connected subpopulation, which is equivalent to broken links due to two-way travel restriction. The time $t\geq T_{0}$.

Until now, the majority of research on metapopulation network models have been devoted to the effect of different network structures, safety-driven one-way travel restrictions, and transmission prediction. Little work has been done on two-way travel restrictions. This paper focuses on the problem of two-way travel restrictions on all neighbor subpopulations, and puts forward a susceptible-infectious-removed (SIR) metapopulation model to study the global transmission of an emerging infectious disease. Using the relative density of the infectious, we define a risk indicator $\rho ~(\rho \in [{0,1}])$ that divides all subpopulations into three categories: risk-free, low-risk, and high-risk. For a subpopulation, if there are no infectious persons, it is a risk-free subpopulation. If the number of infectious persons is larger than 0, and the relative density of infectious persons in the subpopulation is lower than $\rho $, then the subpopulation is a low-risk subpopulation. If the relative density of infectious persons in the subpopulation is no less than $\rho $, it is a high-risk subpopulation. Since the relative infection density of in a subpopulation varies over time, the risk level of the subpopulation varies over time. Let $t_\rho ~(t_\rho \geq 0)$ be the minimal time when a subpopulation goes from low risk to high risk, and $T_{0}~(T_{0}\geq 0)$ be the intervention time on two-way travel restriction. We consider that $T_{0}$ for each subpopulation is the same and that it is independent of the risk level of subpopulations. So the relationship between $T_{0}$ and $t_\rho $ is uncertain. That is, $T_{0}< t_\rho $, $T_{0}>t_\rho $ and $T_{0}=t_\rho $ are all possible. Fig. 1 shows the case of $T_{0}>t_\rho $. Based on the risk indicator $\rho $ and the intervention time on two-way travel restriction $T_{0}$, we define an adaptive metapopulation network (see Fig. 2). An adaptive metapopulation network is a metapopulation network, whose links will be broken if one of the two connected subpopulations is high-risk after $T_{0}$, and then will be reconnected when the high-risk subpopulation becomes low-risk after $T_{0}$. Note that links broken and reconnected on metapopulation networks refer to the link weights, not the underlying network. The adaptive metapopulation network is equivalent to two-way travel restrictions on all neighbor subpopulations. Results show that adaptive metapopulation networks can effectively curb the global spread of infectious diseases.

The paper is organized as follows. In Sec. II, according to the definition of adaptive metapopulation network, an SIR metapopulation model is built. We make a mathematical analysis on this model and calculate the basic reproduction number and final size in Sec. III. In Sec. IV, we simulate the spread of an SIR infectious disease on adaptive metapopulation networks. Conclusions and discussion are given in Sec. V.

## II. Adaptive Metapopulation Network Modeling

In order to investigate the global transmission of an SIR infectious disease under two-way travel restriction on all neighbor subpopulations, we consider a connected adaptive metapopulation network with $n~(n>1)$ nodes, and label its nodes with the elements in a set $\mathbb {V}=\{1,2,\ldots,n\}$. The adjacency matrix $A=(a_{ij})_{n\times n}$ of underlying network is a matrix with entries

\begin{align*} a_{ij}= \begin{cases} 1, & \text {if}~i~\text {is adjacent to} ~j~\textit {and}~i\neq {j}, \\ 0, & \text {otherwise}. \end{cases}\end{align*}

Individuals in each subpopulation are divided into three types, susceptible (S), infectious (I) and removed (R). Removed individuals represent those who have recovered, or died. In an unit time, the susceptible may be infected by the infectious at the transmission rate $\beta $ and become infectious. And an infectious individual is removed (recovered or died) at removed rate $\gamma $. For subpopulation $i$, $S_{i}$, $I_{i}$ and $R_{i}$ are the numbers of susceptible, infectious, removed individuals, respectively. $N_{i}$ is the number of individuals and $N_{i}=S_{i}+I_{i}+R_{i}$. Subpopulations are also divided into four types according to the states of individuals within subpopulations. For subpopulation $i$, if there are only susceptible individuals, i.e., $S_{i}>0, I_{i}=0,\,\, {\text {and}}\,\,R_{i}=0$, it is called risk-free susceptible subpopulation (**FSS**); if the number of infectious individuals equals 0, and the number of removed individuals is larger than 0, i.e., $I_{i}=0,\,\,{\text {and}}\,\,R_{i}>0$, it is called a risk-free recovery subpopulation (**FRS**); if the number of the infectious is greater than 0, and the relative density of the infectious is no less than $\rho $ i.e., $I_{i}>0$, and $I_{i}/N_{i}\geq \rho $, it is called high-risk infectious subpopulation (**HIS**); if $I_{i}>0$, and $I_{i}/N_{i}< \rho $, it is called low-risk infectious subpopulation (**LIS**). **HIS** and **LIS** are collectively referred to as infectious subpopulations (**IS**). Note that the removed individuals in a **FRS** may either be removed from an infectious individual internally, or travel from neighbor subpopulations.

Assuming that individuals travel randomly, we build an ordinary differential equation model as follows

\begin{align*} S'_{i}=&-\beta \frac {S_{i}I_{i}}{N_{i}}-\delta _{i}(t;\rho,T_{0}) S_{i} \\&+\sum \limits _{j=1}^{n}\delta _{j}(t;\rho,T_{0}) a_{ji} w_{ji}(t;\rho,T_{0})S_{j},\tag{1a}\\ I'_{i}=&\beta \frac {S_{i}I_{i}}{N_{i}}-\gamma I_{i}-\delta _{i}(t;\rho,T_{0}) I_{i} \\&+\sum \limits _{j=1}^{n} \delta _{j}(t;\rho,T_{0}) a_{ji} w_{ji}(t;\rho,T_{0})I_{j},\tag{1b}\\ R'_{i}=&\gamma I_{i}-\delta _{i}(t;\rho,T_{0}) R_{i} \\&+\sum \limits _{j=1}^{n} \delta _{j}(t;\rho,T_{0}) a_{ji} w_{ji}(t;\rho,T_{0})R_{j},\quad i\in \mathbb {V}.\tag{1c}\end{align*}

Here $T_{0}$ is the intervention time on two-way travel restrictions (shown in Fig. 1); $\delta _{i}(t;\rho,T_{0})$ represents mobility rate, which depends on the risk indicator $\rho $ and the intervention time for travel restrictions $T_{0}$; square matrix $W=(w_{ij}(t;\rho,T_{0}))_{n\times n}$ is link weights of the adaptive metapopulation network, which also depends on $\rho $ and $T_{0}$. Letting $\Omega _\rho $ be the set of low-risk and risk-free subpopulations, i.e., $\Omega _\rho =\{i\in \mathbb {V} \mid I_{i}/N_{i}< \rho \}$, we have

\begin{align*} \delta _{i}(t;\rho,T_{0})= \begin{cases} \delta, & \text {if}~i ~\in \Omega _\rho ~\text {or}~t< T_{0}, \\ 0, & \text {otherwise}, \end{cases}\end{align*}

and

\begin{align*} w_{ij}(t;\rho,T_{0})= \begin{cases} \dfrac {1}{k_{i}}, & \text {if}~j \in \mathbb {V}_{i}~\text {and} ~t< T_{0}, \\ \dfrac {1}{k^{i\leftrightarrow m}}, & \text {if}~j \in \Omega _\rho ^{i\leftrightarrow m}~\text {and}~t\geq T_{0}, \\ 0, & \text {otherwise}, \end{cases}\end{align*}

where $\mathbb {V}_{i}=\{j\in \mathbb {V}\mid a_{ij}=1\},\,\,k_{i}=|\mathbb {V}_{i}|,\,\,\Omega _\rho ^{i\leftrightarrow m}=\{j\in \Omega _\rho \mid a_{ij}=1,\,\,i\in \Omega _\rho \},\,\,k^{i\leftrightarrow m}=|\Omega _\rho ^{i\leftrightarrow m}|$.

Consider two extreme cases: $\rho =0$ and $\rho =1$. $\rho =0$ is equivalent to the case where the number of infectious individuals is zero. That is, whenever an infectious person is present in a subpopulation, it is high-risk. $I_{i}< 0$ is unreasonable. Thus, when $\rho =0$, there are

\begin{align*} \delta _{i}(t;\rho,T_{0})= \begin{cases} \delta, & \text {if}~I_{i}=0 ~\text {or} ~t< T_{0}, \\ 0, & \text {otherwise}, \end{cases}\end{align*}

and

\begin{equation*}\Omega _{0}^{i\leftrightarrow m}=\{j\in \Omega _{0}\mid a_{ij}=1,~i\in \Omega _{0}\}.\end{equation*}

Obviously, $I_{i}< N_{i}$ for any $i\in \mathbb {V}$ at any time, when $\rho =1$,

\begin{equation*}\delta _{i}(t;\rho,T_{0}) \equiv \delta,\end{equation*}

and

\begin{equation*}\Omega _{1}^{i\leftrightarrow m}=\mathbb {V}_{i}.\end{equation*}

In this case, all **IS** are low-risk, and the two-way travel restriction makes no sense.

From the perspective of controlling and preventing the global spread of an emerging infectious disease, an adaptive metapopulation network is equivalent to a control strategy which limits the two-way travel (abbreviated as **In-Out**). Besides, in order to prevent and control the global spread of an emerging infectious disease, there are two other major one-way travel restrictions. One limits the move-out of individuals in **IS** (abbreviated as **Out**). The other prevents individuals in the neighbor subpopulations of **IS** from traveling to **IS** (abbreviated as **In**). Travel restrictions **In** and **Out** only cut off the one-way movement, while traveling restriction **In-Out** the two-way movement. City (or country) lockdown is equivalent to travel restriction **In-Out**.

For travel restriction **Out**, mobility rates and link weights behave as

\begin{align*} \delta _{i}(t;\rho,T_{0})= \begin{cases} \delta, & \text {if}~i ~\in \Omega _\rho ~\text {or}~t< T_{0}, \\ 0, & \text {otherwise}, \end{cases}\end{align*}

and

\begin{align*} w_{ij}(t;\rho,T_{0})= \begin{cases} \dfrac {1}{k_{i}}, & \text {if}~j ~\in \mathbb {V}_{i},~\text {and} ~i \in \Omega _\rho ~\text {or}~t< T_{0}, \\ 0, & \text {otherwise}. \end{cases}\end{align*}

With regard to travel restriction **In**, mobility rates and link weights become

\begin{equation*}\delta _{i}(t;\rho,T_{0}) \equiv \delta,\end{equation*}

and

\begin{align*} w_{ij}(t;\rho,T_{0})= \begin{cases} \dfrac {1}{k_{i}}, & \text {if} ~j \in \mathbb {V}_{i} ~\text {and} ~t< T_{0}, \\ \dfrac {1}{k^{i\rightarrow m}}, & \text {if}~j \in \Omega _\rho ^{i\rightarrow m} ~\text {and} ~t\geq T_{0}, \\ 0, & \text {otherwise}, \end{cases}\end{align*}

where $\Omega _\rho ^{i\rightarrow m}=\{j\in \Omega _\rho \mid a_{ij}=1\},\,\,k^{i\rightarrow m}=|\Omega _\rho ^{i\rightarrow m}|$.

## III. Mathematical Analysis

In this section, we will calculate the equilibria, the basic reproduction number and the final size of model (1).

### A. Equilibria

On the one hand, summing (1a)–(1c) over $i$, we obtain

\begin{align*} S'=&-\beta \sum \limits _{i=1}^{n}\frac {S_{i}I_{i}}{N_{i}},\tag{2a}\\ I'=&\beta \sum \limits _{i=1}^{n}\frac {S_{i}I_{i}}{N_{i}}-\gamma I,\tag{2b}\\ R'=&\gamma I, \tag{2c}\end{align*}

where $X=\sum _{i=1}^{n} X_{i},\,\,X\in \{S,I,R\}$. Letting the right side of (2) equal to 0, we have

\begin{align*} S'=&-\beta \sum \limits _{i=1}^{n}\frac {S_{i}I_{i}}{N_{i}}=0,\tag{3a}\\ I'=&\beta \sum \limits _{i=1}^{n}\frac {S_{i}I_{i}}{N_{i}}-\gamma I=0,\tag{3b}\\ R'=&\gamma I=0, \tag{3c}\end{align*}

Since for any $i\in \mathbb {V},\,\,I_{i}=0$, according to (3c), $I_{i}=0$ for any $i\in \mathbb {V}$ at equilibria.

On the other hand, summing (1a)–(1c) gives

\begin{align*} N'_{i}&=-\delta _{i}(t;\rho,T_{0}) N_{i} \\&\quad \qquad { +\sum \limits _{j=1}^{n} \delta _{j}(t;\rho,T_{0}) a_{ji}w_{ji}(t;\rho,T_{0})N_{j},\quad i\in \mathbb {V}. } \tag{4}\end{align*}

Define $D=(d_{ij})_{n\times n}$ with entries

\begin{equation*}d_{ij}=\delta _{i}(t;\rho,T_{0})a_{ij}w_{ij}(t;\rho,T_{0}).\end{equation*}

With $\mathbb{N}=[N_{1},\ldots,N_{n}]^{T}$ and $\Lambda =[\delta _{1}(t;\rho,T_{0}),\ldots,\delta _{n} (t;\rho,T_{0})]^{T}$, (4) can be rewritten as

\begin{equation*} \mathsf {N'}=M\,\mathsf{N},\tag{5}\end{equation*}

where $M=D^{T}-{\text {diag}}(\Lambda)$, and ${\text {diag}}(\Lambda)$ is a $n \times n$ matrix, whose diagonal elements forming the vector $\Lambda $.

Since for any $i\in \mathbb {V},\,\,I_{i}=0$ at equilibria, $\delta _{i}(t;\rho,T_{0})\equiv \delta $, $d_{ij}=\delta a_{ij}/k_{i}$, and $\Lambda =[\delta,\ldots,\delta]^{T}$. Note that each column sum of $-M$ is zero, that is, ${\mathsf{1}}_{n}^{T}(-M)=0$, where the $1\times n$ vector ${\mathsf{1}}_{n}^{T}=[1,\ldots,1]$. Thus, matrix $-M$ is a singular M-matrix. From (5), letting $N=\sum _{i=1}^{n} N_{i}$, we obtain that the total population $N$ is constant (because $N'=0$). Subject to this constraint, by Theorem 3.3 in [13], we show that (5) has a unique positive equilibrium $N_{i}=N_{i}^{*}$, which is globally asymptotically stable.

Accordingly, for any $i\in \mathbb {V},\,\,I_{i}=0$ and $S_{i}+R_{i}=N_{i}^{*}$ at equilibria. If for all $i$, $R_{i}=0$, the equilibrium is a disease-free equilibrium; otherwise we call the equilibrium as epidemic equilibrium.

Next, we calculate the basic reproduction number.

### B. The Basic Reproduction Number

We calculate the basic reproduction number $R_{0}$ following the approach of van den Driessche and Watmough [14]. $R_{0}$ indicates the number of people infected by an infectious individual during his average period of illness at the beginning of the disease, when all are susceptible. Obviously, there exists a disease-free equilibrium $E^{0}=(N_{1}^{*},\ldots,N_{n}^{*},\underbrace {0,\ldots,0}_{2n})$ for (1). According to (1), the rate of appearance of new infections $F$ and the rate of transfer of individuals out of the compartments $V$ in the $E^{0}$ are given by

\begin{equation*} F={\text {diag}}(\beta)\end{equation*}

and

\begin{equation*} V={\text {diag}}(\gamma)+{\text {diag}}(\Lambda)-D^{T},\end{equation*}

here $F$ and $V$ are $n\times n$ matrices. Using the next-generation matrix theory [14], the basic reproduction number is $R_{0}=\rho (FV^{-1})$, where $\rho $ is the spectral radius of the matrix $FV^{-1}$.

In the following, we calculate $R_{0}$. Note that the sum of each column of matrix $V$ is $\gamma $ and the matrix $V$ is column diagonally dominant. So $V$ is an irreducible nonsingular M-matrix. Thus $V^{-1}$ is a positive matrix.

Matrix $V$ has column sum $\gamma $, i.e., ${\mathsf{1}}_{n}^{T} V=\gamma {\mathsf{1}}_{n}^{T}$. Hence ${\mathsf{1}}_{n}^{T}V^{-1}=1/\gamma {\mathsf{1}}_{n}^{T}$. Therefore, ${\mathsf{1}}_{n}^{T} FV^{-1}=\beta /\gamma {\mathsf{1}}_{n}^{T}$, that is, matrix $FV^{-1}$ has column sum $\beta /\gamma $. By Theorem 1.1 in chapter 2 in [15], the basic reproduction number is

\begin{equation*} R_{0}=\frac {\beta }{\gamma }.\end{equation*}

Obviously, $R_{0}$ depends only on disease parameters $\beta $ and $\gamma $, rather than on mobility rate and network structure. What is more, $R_{0}$ equals to the basic reproduction number $R_{0}^{i}$ for each subpopulation $i$ when there is no travel. This is because that transmission rate $\beta $ and removed rate $\gamma $ for each subpopulation $i$ keep the same.

### C. Final Size

For each subpopulation $i\in \mathbb {V}$, given initial conditions $N_{i}=N_{i}^{*}$, and without consideration of travel restriction, dividing (2a) by (2c), we have

\begin{equation*} \frac {dS}{dR}=-\frac {\beta }{\gamma }\sum \limits _{i=1}^{n}\frac {S_{i}I_{i}}{N_{i}^{*}I}.\tag{6}\end{equation*}

On the one hand,

\begin{align*} \frac {dS}{dR}\leq&-\frac {\beta }{\gamma }\frac {1}{N^{max}}\sum \limits _{i=1}^{n}\frac {S_{i}I_{i}}{I} \\< &-\frac {\beta }{\gamma }\frac {1}{N^{max}}S, \tag{7}\end{align*}

where $N^{max}$ is the maximum of $N_{i}^{*},~i\in \mathbb {V}$. Dividing the right side of (7) to the left side, multiplying $dR$ to the right side, and integrating both sides we obtain

\begin{equation*} R(\infty)< \frac {N^{max}}{R_{0}}({\text {ln}} S(0)-{\text {ln}} S(\infty)).\tag{8}\end{equation*}

Here $R(\infty)$ is the final size of epidemic, $S(0)$ is the initial susceptible individuals in the metapopulation network, $S(\infty)$ gives the number of susceptible individuals who escape the epidemic, and $S(\infty)+R(\infty)=N$. On the other hand,

\begin{equation*} \sum \limits _{i=1}^{n}\frac {S_{i}I_{i}}{N_{i}^{*}I}\leq \frac {1}{N^{min}}\sum \limits _{i=1}^{n}\frac {S_{i}I_{i}}{I}< \frac {1}{N^{min}}S,\end{equation*}

so

\begin{equation*} \frac {dS}{dR}>-\frac {\beta }{\gamma }\frac {1}{N^{min}}S.\tag{9}\end{equation*}

Here $N^{min}$ is the minimum of $N_{i}^{*},~i\in \mathbb {V}$. Similar to (7), we have

\begin{equation*} R(\infty)>\frac {N^{min}}{R_{0}}({\text {ln}} S(0)-{\text {ln}} S(\infty)).\tag{10}\end{equation*}

Therefore, without consideration of travel restriction, the final size satisfies

\begin{align*} \frac {N^{min}}{R_{0}}({\text {ln}} S(0)-{\text {ln}} S(\infty))< &R(\infty)\qquad \\< &\frac {N^{max}}{R_{0}}({\text {ln}} S(0)-{\text {ln}} S(\infty)).\tag{11}\end{align*}

When $\rho =0$, and $T_{0} =0$, infectious disease will outbreak only in the initial infectious subpopulation, not globally. Labeling the initial infectious subpopulation by $i_{0}$, we have

\begin{align*} S_{i_{0}}'=&-\beta \frac {S_{i_{0}}I_{i_{0}}}{N_{i_{0}}^{*}},\tag{12a}\\ I_{i_{0}}'=&\beta \frac {S_{i_{0}}I_{i_{0}}}{N_{i_{0}}^{*}}-\gamma I_{i_{0}},\tag{12b}\\ R_{i_{0}}'=&\gamma I_{i_{0}}. \tag{12c}\end{align*}

With regard to other subpopulations in the adaptive metapopulation network, there is no infection, just the susceptible’ mobility among susceptible subpopulations. So the final size of whole network equals to the final size of infectious subpopulation $i_{0}$. Dividing (12c) by (12a) we have

\begin{equation*} dR_{i_{0}}=-\frac {\gamma N_{i_{0}}^{*}}{\beta }\frac {dS_{i_{0}}}{S_{i_{0}}}.\end{equation*}

Integrating both sides we obtain

\begin{equation*} R(\infty)=R_{i_{0}}(\infty)=\frac {N_{i_{0}}^{*}}{R_{0}}({\text {ln}} S_{i_{0}}(0)-{\text {ln}} S_{i_{0}}(\infty)).\tag{13}\end{equation*}

Here $S_{i_{0}}(0)$ and $S_{i_{0}}(\infty)$ are the numbers of initial susceptible individuals and susceptible individuals who escape the disease in subpopulation $i_{0}$, respectively.

It is difficult to compare Eqs. (11) and (13) in theory. In the next section, we will simulate these two cases and compare their final sizes.

## IV. Monte Carlo Simulation Results

In this section, we simulate an SIR infectious disease on two kinds of adaptive metapopulation networks with the same average degree $\langle k\rangle =6$, and average population $\overline {N}=1000$. The generation of metapopulation networks is following Molloy and Reed algorithm [17]. Parameters $\beta =0.4$, $\gamma =0.2$ and $\delta =0.1$. For subpopulation $i$, its initial population is

\begin{equation*}N_{i}^{0}=\frac {k_{i}}{\langle k\rangle }\overline {N},\end{equation*}

where $k_{i}$ is the degree of subpopulation $i$. All values in the figures of this section are obtained by averaging over 100 stochastic realizations.

### A. The Comparison of One-Way and Two-Way Travel Restrictions

In this subsection, we compare the impact of three kinds of travel restrictions: **In-Out**, **In** and **Out**, on the global spread of infectious diseases.

**FIGURE 3.**
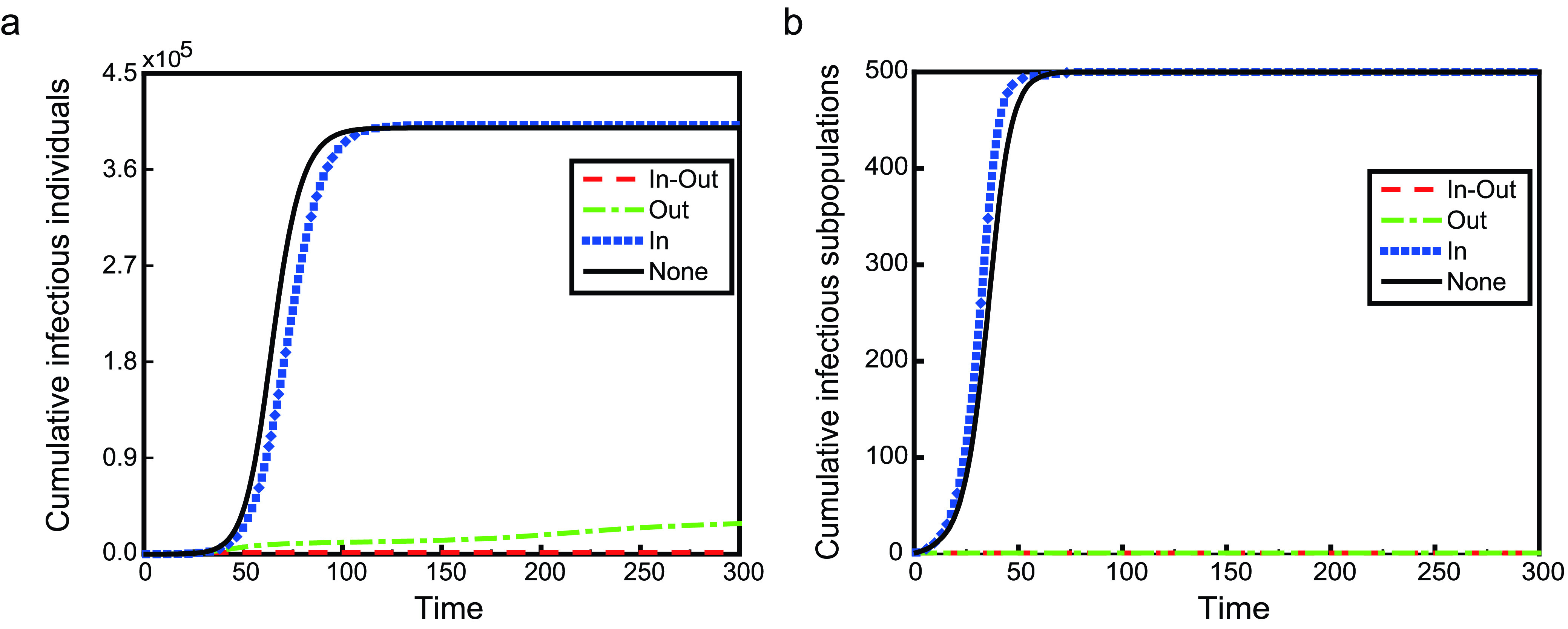
The time evolution of the number of cumulative infectious individuals and subpopulations on a poisson metapopulation network. Control strategies are In-Out (red dashed lines), Out (green dot-dashed lines), In (blue dot lines), and None (black solid lines), individually. The panel a shows the number of cumulative infectious individuals evolving over time, while the panel b is the number of cumulative infectious subpopulations. Parameter $T_{0}=0$.

**FIGURE 4.**
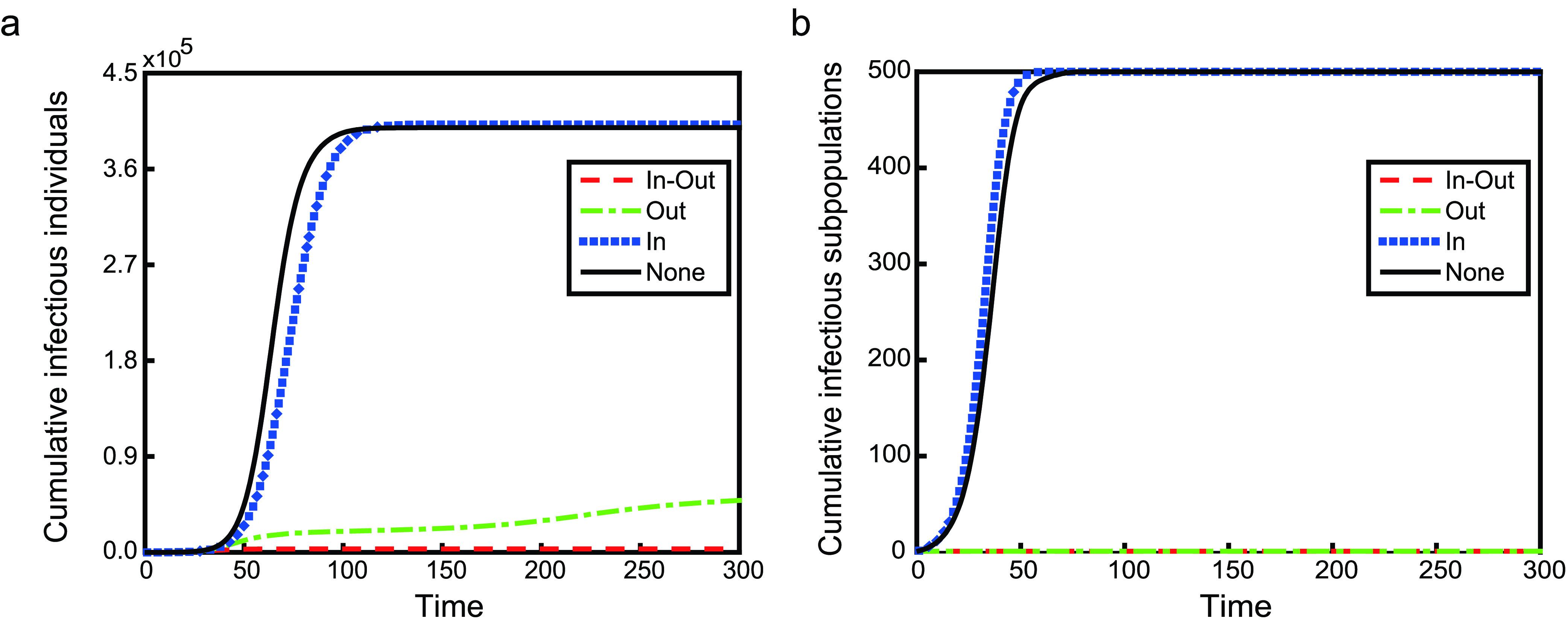
The time evolution of the number of cumulative infectious individuals and subpopulations on a power-law metapopulation network. Others are similar to Fig. 3.

As shown in Figs. 3 and 4, we simulate an SIR transmission process on two kinds of metapopulation network with 500 subpopulations under three kinds of travel restrictions. Fig. 3 is the case on a poisson metapopulation network, and Fig. 4 is the case on a power-law metapopulation network. In these two figures, the legend **None**, a reference, shows the case without any travel restriction or the case where $\rho =1$. For travel restrictions **In-Out**, **In** and **Out**, $\rho =0$. The left panels show the number of cumulative infectious individuals evolving over time, while the right panels are the number of cumulative infectious subpopulations. There are five infectious individuals in a subpopulation with the maximum degree in the initial time.

**FIGURE 5.**
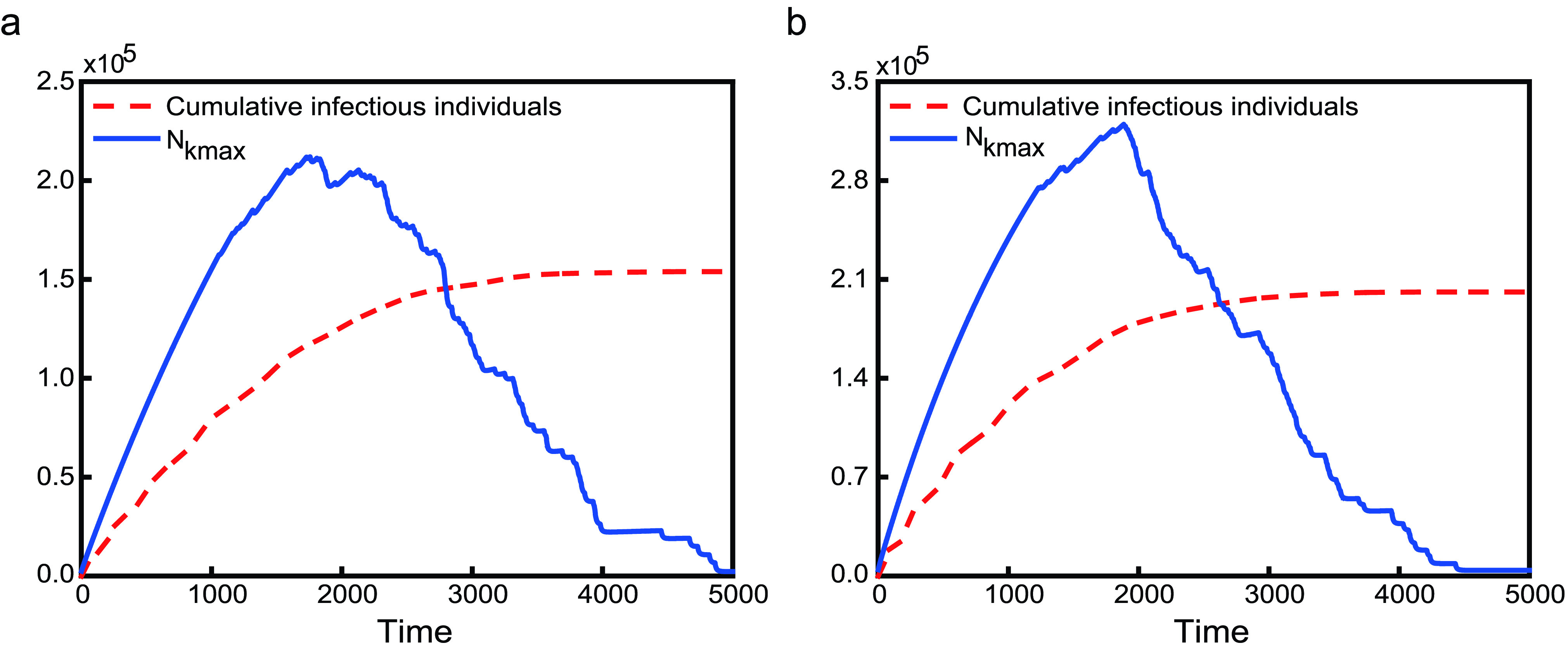
The time evolution of the number of cumulative infectious individuals and the population of the subpopulation with the maximum degree under restriction **Out** on metapopulation networks. They are illustrated by red dashed lines and blue solid lines, individually. The left panel shows the case of poisson network, while the right panel is of power-law degree distribution.

Among three travel restrictions, **In** is the worst, because it accelerates the global spread of infectious diseases, not only the speed of global transmission but also the final size. Although the number of cumulative infectious subpopulations for travel restrictions **In-Out** and **Out** are both one, the situation is quite different. Travel restriction **In-Out** can effectively curb the global spread of infectious diseases, and the disease will not spread to other individuals in other subpopulations. The final size of **In-Out** is less than 1% of that of **None**. In the short term, both travel restrictions are of benefit to preventing the global spread of infectious diseases. However, in the long term, the infectious disease will globally spread in the sense of the number of cumulative infectious individuals under the case of travel restriction **Out** (shown as red dashed lines in Fig. 5). Nevertheless, the final size of **Out** is about 60% lower than that of **None**. Therefore, preventing individuals in the infectious subpopulations from entering their neighbors is also a relatively effective prevention and control measure of global transmission.

Why is only controlling one-way movement not the best measure of suppressing the global spread of disease? This is due to the fact that the mobility between subpopulations with infectious individuals and without is asymmetric. In theory, for the subpopulation with the maximum degree (labeled by $k_{max}$), when there exists(exist) infectious individual(s), the dynamical equations for its population are

**FIGURE 6.**
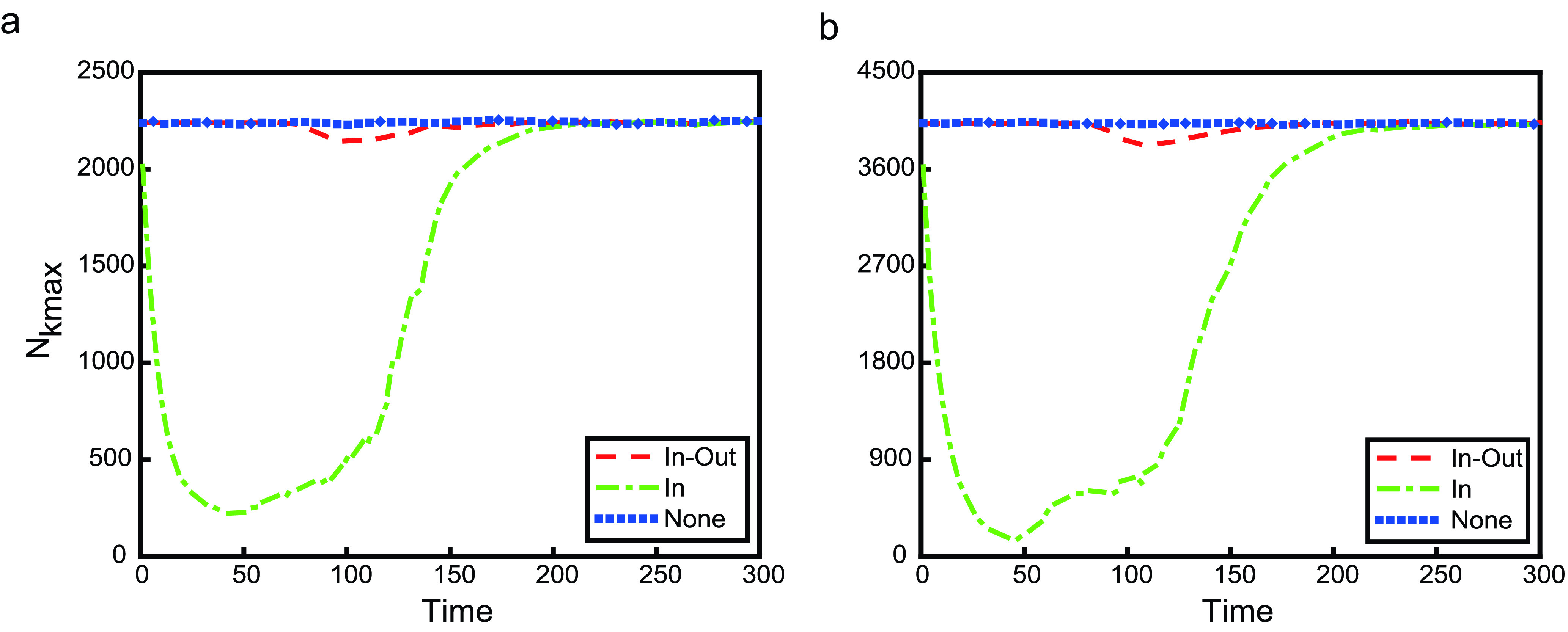
The time evolution of $N_{k_{max}}$. Three curves are **None** (blue dot line), **In-Out** (red dashed line), and **In** (green dot-dashed line), individually. The left panel shows the case of poisson network, while the right panel is of power-law degree distribution.

\begin{align*} \mathbf {None}:~N'_{k_{max}}=&-\delta N_{k_{max}}+ \sum \limits _{j=1}^{n} \delta a_{j,k_{max}}w_{j,k_{max}}N_{j}, \tag{14}\\ \mathbf {In}:~N'_{k_{max}}=&-\delta N_{k_{max}}, \tag{15}\\ \mathbf {Out}:~N'_{k_{max}}=&\sum \limits _{j=1}^{n} \delta _{j}(t;\rho,T_{0}) a_{j,k_{max}}w_{j,k_{max}}(t;\rho,T_{0})N_{j}, \\ \tag{16}\\ \mathbf {In-Out}:~N'_{k_{max}}=&0,\tag{17}\end{align*}

respectively. Obviously, the difference between **In** and **None** is that there is no any individuals moving from the neighbor subpolulation(s) of subpolulation $k_{max}$ to itself. However, **In** does not restrict the mobility of infectious individuals, which leads to more susceptible subpopulations being infected. Although travel restrictions **In-Out** and **Out** can effectively prevent infectious individuals in infectious subpopulations from entering to their neighbor subpopulations, they are different. For **In-Out**, there is no any individuals moving between subpolulation $k_{max}$ and its neighbor subpolulation(s). While for **Out**, susceptible individuals go from the neighbor subpolulation(s) of subpolulation $k_{max}$ to itself until $I_{k_{max}}\equiv 0$, which leads to more susceptible individuals being infected in subpopulation $k_{max}$. Travel restrictions **Out** and **In** break the balance of the mobility. Under the case of travel restriction **Out**, for subpopulation $k_{max}$, susceptible individuals from its neighbor subpopulations travel to it; susceptible individuals from its second neighbor subpopulation(s) travel to its neighbor subpopulations first and then travel to subpopulation $k_{max}$; susceptible individuals in the third neighbor subpopulation(s) will transfer the second neighbor(s) and the neighbors will eventually enter subpopulation $k_{max}$; and so on, in the end, the majority of individuals in the network will move into subpopulation $k_{max}$, causing widespread infection. But this process takes a long time. When $I_{k_{max}}=0$, individuals in subpopulation $k_{max}$ travel to other subpopulations. With regard to travel restriction **In**, the continuous move-out of individuals from infectious subpopulation results in a decreasing number of individuals until there are no infectious individuals. In Figs. 5 and 6, we plot the time series of $N_{k_{max}}$. The simulation results are consistent with the theoretical analysis.

From what has been discussed above, adaptive metapopulation networks can prevent effectively the global transmission of infectious diseases.

### B. The Threshold of Risk Indicator

**FIGURE 7.**
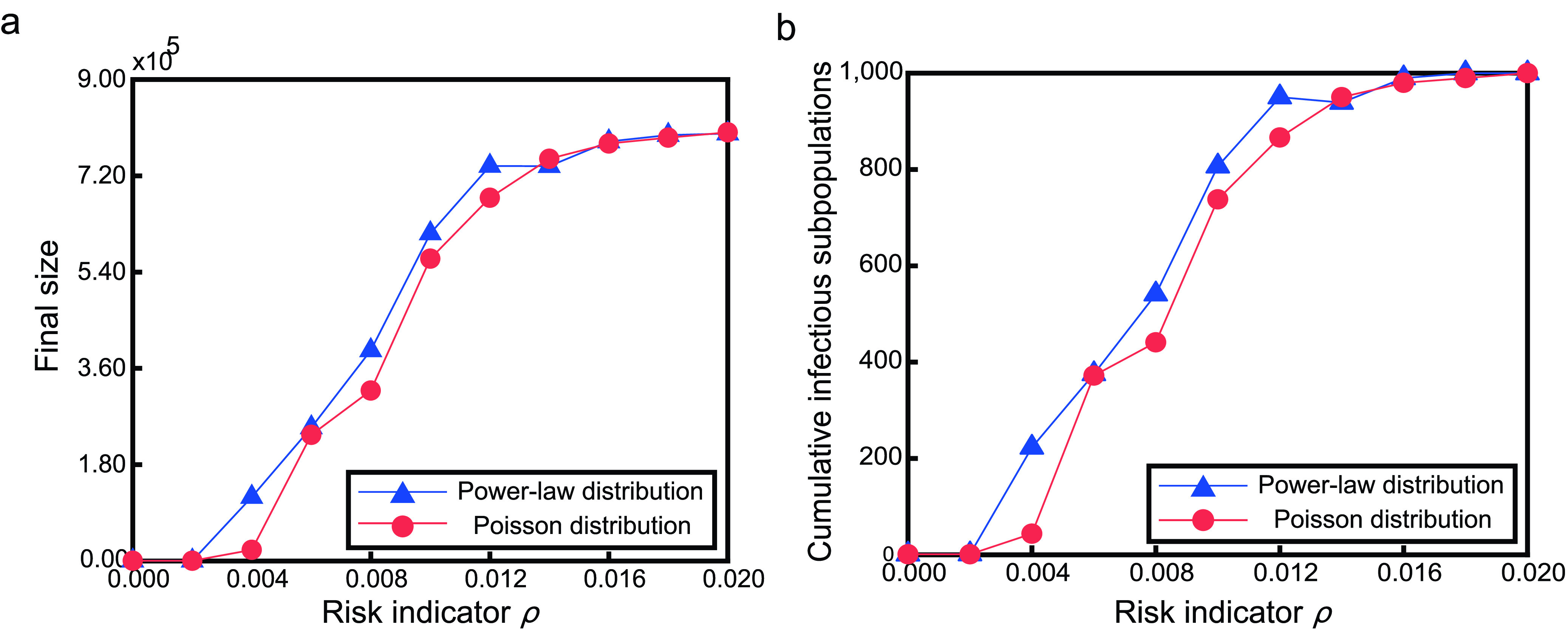
The final size and the number of cumulative infectious subpopulations under different $\rho $. Figures are under two kinds of different adaptive metapoplation networks, red circles for poisson degree distribution and blue triangles for power-law degree distribution. The left panel shows the final size versus $\rho $, while the right panel is the number of cumulative infectious subpopulations. Parameter $T_{0}=0$.

The mobility of individuals is sometimes necessary to promote economic and cultural globalization. Moreover, human behaviour is often out of control. So $\rho =0$ is too ideal. However, $\rho =1$ is equivalent to the case of no travel restriction. It is more appropriate that $\rho \in (0,1)$. What is the threshold of risk indicator $\rho $ for preventing the global spread of disease? As illustrated in Fig. 7, we simulate the final size and the number of cumulative infectious subpopulations under different $\rho $. We consider two kinds of adaptive metapoplation networks with 1000 subpopulations: poisson degree distribution (red circles) and power-law degree distribution (blue triangles). In the initial time, there are five infectious individuals in a randomly chosen subpopulation with a minimal degree.

For these two kinds of networks, the infectious disease spread globally when $\rho \geq 0.004$. In the case of $\rho < 0.004$, there are little infectious individuals moving from the initial infectious subpopulation to its neighbors before being removed. When $\rho \geq 0.004$, as $\rho $ increases, the number of move-out of infectious individuals in infectious subpopulation(s) increases. So the number of infectious subpopulations goes up, and the final size increases. For the same $\rho $, the speed of global transmission in power-law networks is faster than that in poisson networks. This is due to the heterogeneity of the network structure. That is to say, the heterogeneity of the network structure promotes the global spread of infectious diseases.

### C. The Effect of Intervention Time

**FIGURE 8.**
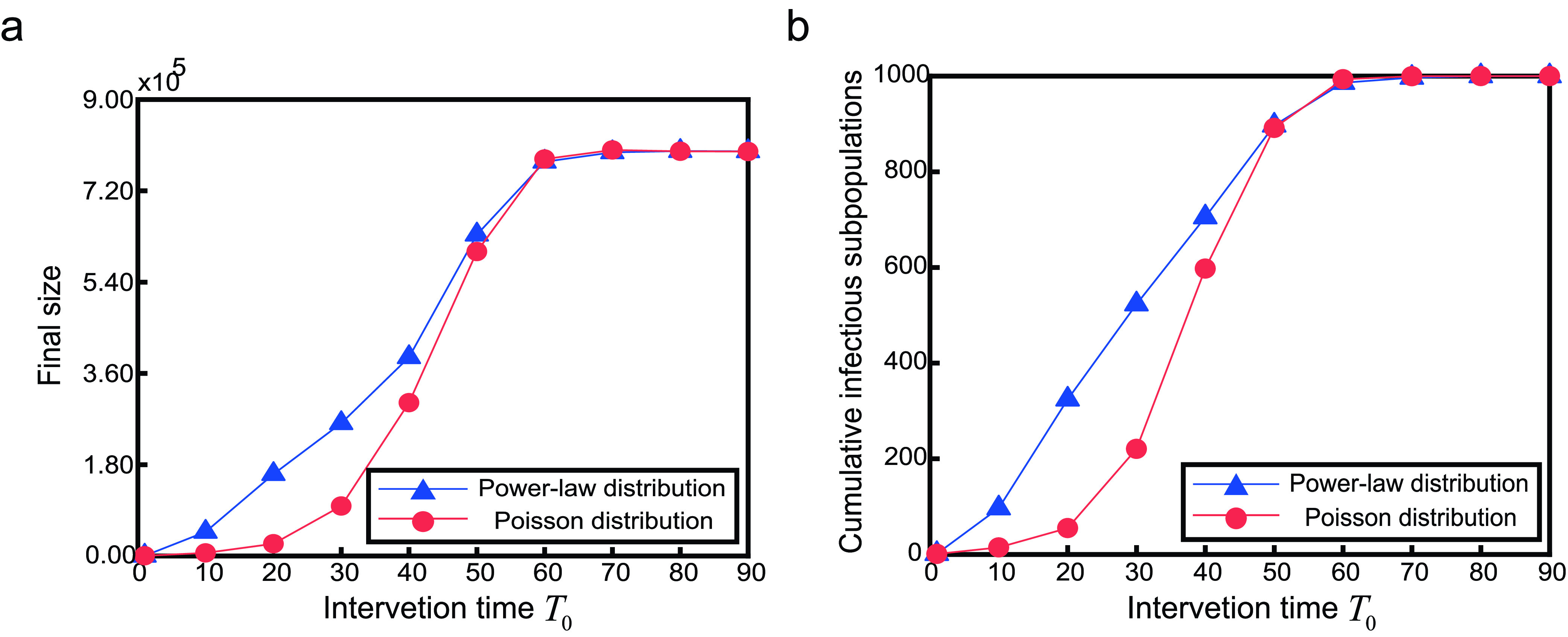
The effect of intervention time $T_{0}$ on final size and cumulative infectious subpopulations. Parameter $\rho =0.002$.

In real life, the response to an emerging infectious disease tends to lag. This implies a delay between the onset of disease transmission and the onset of two-way travel restrictions. Fig. 8 displays the effect of intervention time for two-way travel restrictions $T_{0}$ on both the final size and the number of cumulative infectious subpopulations under two kinds of metapopulation networks.

Obviously, the earlier $T_{0}$, the better to prevent the global spread of the infectious diseases. The first ten days since the onset of infectious disease are of great importance. The speed of global transmission is relatively slow, and implementing two-way travel restrictions during this period will be effective in preventing the global spread of the disease. When $T_{0}\geq 60$, the existence of adaptive metapopulation network makes no sense, i.e., two-way travel restrictions do not work. Similar to Fig. 7, for the same $T_{0}$, final size and the number of cumulative infectious subpopulations of power-law networks are larger than those of poisson networks. This also indicates that the heterogeneity of the network structure promotes the global spread of infectious diseases.

## V. Conclusion and Discussion

Adaptive metapopulation networks help suppress the global transmission of an acute emerging infectious disease. This paper defined a risk indicator $\rho $ according to the relative infection density and divided subpopulations into three types: risk-free, low-risk, and high-risk. Based on the risk indicator and intervention time for two-way travel restrictions $T_{0}$, this essay defined an adaptive metapopulation network and proposed an SIR metapopulation model on this network to investigate the issue of two-way travel restrictions on all neighbor subpopulations. Then this paper obtained the equilibria, at which there are no infectious individuals; the basic reproduction number, which is independent of mobility rate; and the final size under special cases. Finally, this paper presented Monte Carlo simulation results on two kinds of metapopulation networks with different degree distributions but the same average degree. Comparing two-way travel restriction and two kinds of one-way travel restrictions, we find that controlling the movement of infectious individuals helps prevent the global spread of infectious diseases. The conclusion is obvious. However, the mobility of susceptible individuals also makes for the transmission of disease. Therefore, adaptive metapopulation networks under two-way travel restriction help prevent the global spread of diseases. Furthermore, we obtained that the threshold of risk indicator $\rho $ is 0.004. When $\rho \geq 0.004$, infectious diseases will spread globally. What is more, the earlier the intervention time for two-way travel restriction $T_{0}$ is, the better to prevent the disease from spreading globally. It is better to implement two-way travel restrictions during the first ten days since the onset of the disease. When $T_{0}\geq 60$, two-way travel restriction makes no sense. In addition, simulation results show that the heterogeneity of the network structure promotes the global spread of infectious diseases.

Our results will provide some useful insights on the global transmission prevention and control of an emerging acute infectious disease. Timely two-way travel restrictions (or city lockdown) help suppress the global spread of the epidemic. However, for lockdown subpopulation, controlling the spread of disease remains a major challenge. Adequate medical resources are needed to quarantine close contacts with the infectious, reduce the time that takes for infectious persons to be diagnosed, and explore effective treatments. Even if two-way travel restriction can not be implemented, the mobility of infectious individuals should be restricted. In order to prevent the invasion of infectious individuals, there are a series of control measures. Persons entering the subpopulation shall be quarantined. Individuals leaving the subpopulation need to provide a health certificate and register their origin and destination information. In addition, it is necessary to reduce the number of flights. Within an infectious subpopulation, control measures, including isolating the infectious, quarantining close contacts, keeping social distance, and so on, should be taken to mitigate infections. For an emerging infectious disease, there have been many studies on control measures within subpopulations [18]–[20]. In this article, we mainly focused on the prevention and control of the global spread of the epidemic, so we did not consider the control measures within a subpopulation.

Although we have investigated the issue of two-way travel restriction on all neighbor subpopulations, there are still some problems in this paper to be further solved.

• Travel restrictions we considered is on all neighbor subpopulations. Because of subpopulation differences, travel restrictions might be only on some neighbor subpopulations.
• With regard to the intervention time on travel restrictions, we consider it is the same for all subpopulations. In fact, the disease onset time of each subpopulation is often different, and the intervention time may vary. So intervention time for each subpopulation may be the sum of its disease onset time and a delay.
• In this paper, we only focused on the effect of two-way travel restrictions on the global spread of infectious diseases, ignoring its effect on each subpopulation. In the future, we will consider both the global and local impact of prevention and control measures. Besides, we will study the global and local impact of travel restrictions, keeping social distance, isolating the infectious and quarantining close contacts at the same time on global transmission prevention and control.
